# Challenges to complete and useful data sharing

**DOI:** 10.1186/s13063-017-1816-8

**Published:** 2017-02-14

**Authors:** Lawrence Mbuagbaw, Gary Foster, Ji Cheng, Lehana Thabane

**Affiliations:** 1 Department of Health Research Methods, Evidence and Impact, McMaster University, Hamilton, ON Canada; 2 Biostatistics Unit, Father Sean O’Sullivan Research Centre, The Research Institute, St Joseph’s Healthcare Hamilton, Hamilton, ON Canada; 3 Centre for Development of Best Practices in Health (CDBPH), Yaoundé Central Hospital, Yaoundé, Cameroon; 4 Departments of Paediatrics and Anaesthesia, McMaster University, Hamilton, ON Canada; 5 Centre for Evaluation of Medicine, St Joseph’s Healthcare Hamilton, Hamilton, ON Canada; 6 Population Health Research Institute, Hamilton Health Sciences, Hamilton, ON Canada

**Keywords:** Data sharing, Fairness, Remote access, Jurisdiction, Programmatic, Multicentre

## Abstract

Data sharing from clinical trials is one way of promoting fair and transparent conduct of clinical trials. It would maximise the use of data and permit the exploration of additional hypotheses. On the other hand, the quality of secondary analyses cannot always be ascertained, and it may be unfair to investigators who have expended resources to collect data to bear the additional burden of sharing. As the discussion on the best modalities of sharing data evolves, some of the practical issues that may arise need to be addressed. In this paper, we discuss issues which impede the use of data even when sharing should be possible: (1) multicentre studies requiring consent from all the investigators in each centre; (2) remote access platforms with software limitations and Internet requirements; (3) on-site data analysis when data cannot be moved; (4) governing bodies for data generated in one jurisdiction and analysed in another; (5) using programmatic data collected as part of routine care; (6) data collected in multiple languages; (7) poor data quality. We believe these issues apply to all primary data and cause undue difficulties in conducting analysis even when there is some willingness to share. They can be avoided by anticipating the possibility of sharing any clinical data and pre-emptively removing or addressing restrictions that limit complete sharing. These issues should be part of the data sharing discussion.

## Background

In the past few years, we have experienced an upsurge in calls for complete sharing of data from clinical trials and other primary studies. Many important steps have been taken in this direction, including the opening of data sharing repositories [1], mandatory sharing of data by journals as proposed by the International Committee of Medical Journal Editors (ICMJE) [2], pharmaceutical companies making their data available [3] and published guidance on how to share data [4].

Data sharing comes with the advantages of maximising the use of data collected as an increased tribute to study participants, exploring further bits of evidence that might not have been part of the initial investigation and providing the ability to merge data from individual studies [5, 6]. It also serves to maintain integrity in data analysis in a situation where there is a concern about a conflict of interest [6]. In addition, data sharing can reduce duplication of research efforts and costs as well as patient exposure to potentially harmful interventions in new trials, and it can enhance the decision-making process from regulatory, guideline and clinical perspectives [4].

The ideal of complete data sharing is hampered by many concerns and challenges. Some concerns include the misinterpretation of the data by a secondary user, inappropriate merging of heterogeneous datasets and ’parasitic’ data use — with the sole aim of stealing research productivity [5]. There is also the risk of breaches of confidentiality and the burden of administrative and financial costs associated with data sharing [4]. More so, if data are shared immediately after a trial is completed, there is a risk of the original investigators not having sufficient time to publish the primary results of the trial and any additional secondary analyses [7].

While the focus of data sharing relates to clinical trials, there are other methodologies for which data sharing may be beneficial. A good example is the use of a new pharmaceutical drug in development that has been approved for a compassionate care programme. Data collected within such a programme will be useful in providing evidence on the merits of the drug.

In this paper, we share the experiences of the Biostatistics Unit of The Research Institute at St Joseph’s Healthcare Hamilton. We describe situations that cause delays in the data acquisition process. Details on the actual studies in which these issues occurred are not revealed.

## Approval from all authors is required for some multicentre studies

In certain instances, it may be challenging to obtain data from a multicentre study because all the principal investigators (PIs) of each site are expected to provide consent. This can lead to enormous delays in the data sharing process, especially in multinational trials including many sites. This problem can be resolved by anticipating data sharing requirements prior to initiating the study, so that the individual PIs can consent to the possibility of sharing their data. Seeking consent from each individual site is akin to looking up patients to request their consent for secondary data analysis after a trial is complete. It renders the process unduly onerous and should be discouraged. Steps should be taken at the outset of the study to anticipate future data sharing.

## Remote platforms

Data sharing via remote platforms offers researchers the possibility of analysing a dataset from a remote desktop through a secure connection without the possibility of downloading the data. With this approach, the repository is a 'trusted middle-man’. While this ensures the safety of the data and encourages transparency, it causes several hindrances. First, a strong and reliable Internet connection is required to stay connected to the secure platform. Second, any data merging will require that any other datasets be put onto that secure platform, even though typical data sharing agreements preclude sharing of data with a third party. Finally, remote platforms may not have all the software an analyst would require to clean, prepare and analyse the data.

## Data analysis on site

Requiring that data be analysed on site implies that the analyst should be physically present at a specific terminal in a building to have access to the data. Long-distance travel, accommodation and travel risk are some of the reasons why this kind of data sharing is not pragmatic. In addition, one would have to physically transport other datasets to this specific site. It also creates challenges for re-analyses and further explorations of data.

## Governing bodies

Given that the legislation on data varies in different parts of the world, standards should be set with regard to which jurisdiction is responsible for implementing the terms of data sharing agreements. Understandably, each party would prefer to be governed by the legislations of the jurisdiction in which they reside. This is often not acceptable to second parties.

## Securing ethics approval for programmatic data

If one requests data that have not been collected as part of research (therefore, formal consent was not obtained) but rather as part of routine care, who is responsible for securing ethics approval for data sharing? It may be unfair to request that the custodians of the data initiate the potentially burdensome process of getting ethics approval to share data, but on the other hand it would also be challenging to prepare, submit and follow up a request for ethics approval in a distant country, especially if there are language differences.

## Data collected in multiple languages

For some multicentre studies, data text fields are completed in the local language and require considerable time and effort to translate. Analysts may find themselves having to deal with three or four languages in the same dataset. At the very minimum, data should be collected and coded in only one language.

## Data quality

Given the time and effort required to clean and prepare a dataset for analysis, investigators often share data that have not been cleaned. This may lead to incorrect values being used for analysis and, consequently, potentially misleading results. We believe the persons who created the dataset are the best equipped to clean it, as they have a better understanding of the context of the study, why values may be missing and how best to replace them.

## Conclusion

Data collected for research or otherwise should have data sharing arrangements in place prior to collecting the data for other potential users. Remotely provided data or data requiring travel are not completely shared, in our opinion, and place undue duress on the analyst. The instances described above are situated somewhere along the continuum of absolutely no data shared, on the one hand, to a clean and ready-for-analysis dataset on the other. We have enormous appreciation for the efforts researchers are making for others to be able to access and use their data, but we believe more can be done. Now is the time to seriously consider the practical modalities of data sharing, not only for clinical trials, but for all clinical studies.
